# Contactless measurements of photocarrier transport properties in perovskite single crystals

**DOI:** 10.1038/s41467-019-09538-7

**Published:** 2019-04-08

**Authors:** Xiwen Gong, Ziru Huang, Randy Sabatini, Chih-Shan Tan, Golam Bappi, Grant Walters, Andrew Proppe, Makhsud I. Saidaminov, Oleksandr Voznyy, Shana O. Kelley, Edward H. Sargent

**Affiliations:** 1 Department of Electrical and Computer Engineering, University of Toronto, 35 St. George Street, Toronto, ON M5S 1A4 Canada; 2 Department of Chemistry, University of Toronto, 80 St. George Street, Toronto, ON M5S 1A4 Canada; 3 Department of Pharmaceutical Sciences, Leslie Dan Faculty of Pharmacy, University of Toronto, Toronto, ON M5S 3M2 Canada

## Abstract

The remarkable properties of metal halide perovskites arising from their impressive charge carrier diffusion lengths have led to rapid advances in solution-processed optoelectronics. Unfortunately, diffusion lengths reported in perovskite single crystals have ranged widely – from 3 μm to 3 mm – for ostensibly similar materials. Here we report a contactless method to measure the carrier mobility and further extract the diffusion length: our approach avoids both the effects of contact resistance and those of high electric field. We vary the density of quenchers – epitaxially included within perovskite single crystals – and report the dependence of excited state lifetime in the perovskite on inter-quencher spacing. Our results are repeatable and self-consistent (i.e. they agree on diffusion length for many different quencher concentrations) to within ± 6%. Using this method, we obtain a diffusion length in metal-halide perovskites of 2.6 μm ± 0.1 μm.

## Abstract (web summary)

Reliable and consistent measurement of the carrier diffusion length in metal halide perovskite single crystals has proven difficult. Here Gong, Huang et al. systematically included very dilute quantum dot quenchers into perovskite crystals and develop a contactless and self-consistent method for the task.

## Introduction

Solution-processed photovoltaic materials have facilitated the emergence of third generation solar cell technology with low cost, high efficiency, and flexibility. Thin film solar cells employing metal-halide perovskites have achieved an exceptional 23.7% certified power conversion efficiency (PCE)^1^. In order to reach high PCEs, the active layers must achieve an extended diffusion length (*L*_d_) **–** the distance over which the limiting photocarrier diffuses before it recombines^2,3^.

Single crystals are an ideal platform for studying the ultimate potential of a material, for they are not limited by morphology and grain boundaries. The electrical properties of halide perovskite single crystals have been investigated extensively in recent years, with charge carrier mobilities, *L*_d_, and trap densities reported using a variety of methods.

By probing the photocurrent in solar cell devices, or by combining space charge limited current measurements with optical measurements, researchers have reported electron diffusion lengths inside perovskite crystals. The range has been between 175 μm and 3 mm^4^ using these approaches. In contrast, Shi et al. reported L_d_ using mobility measured from electrical methods such as time-of-flight (TOF) transient photocurrent and Hall effect measurement^5^; when they combined the drift mobility with the photocarrier lifetime obtained from transient photoluminescence, the authors estimated the diffusion length of MAPbBr_3_ perovskite single crystal to reside in the range between 3 to 17 μm.

One of the challenges that may explain these order-of-magnitude discrepancies is that Hall effect, TOF, and SCLC all probe mobilities near the respective Fermi levels during the experiment – and the (nonequilibrium, high-injection-level) Fermi level is widely different in each case. As a result, different mobility-limiting trap densities and depths are sampled. PL lifetime is acquired under a different set of conditions involving low-level or high-level injection, depending on the excitation pulse intensity. The quasi-Fermi level of the relevant charge carrier in the PL component of these studies may differ from that during the electronic injection based transport studies^6^.

Contactless methods including transient microwave conductivity (TRMC) and transient terahertz conductivity (THz) spectroscopy have been utilized to study the transport properties of perovskite thin films and single crystals^7–21^. These methods are not widely available in the average optoelectronic materials research lab; whereas transient absorption and especially photoluminescence are more broadly disseminated (Supplementary Table 1).

We pursued the development of a method to measure diffusion lengths of perovskite single crystal directly and straightforwardly. The technique is based on a 3D diffusion-quenching method^22,23^: it measures the rate at which the nonequilibrium carrier distribution is diminished by spatial diffusion toward luminescent reporters epitaxially embedded in the perovskite.

## Results

**Fig. 1:**
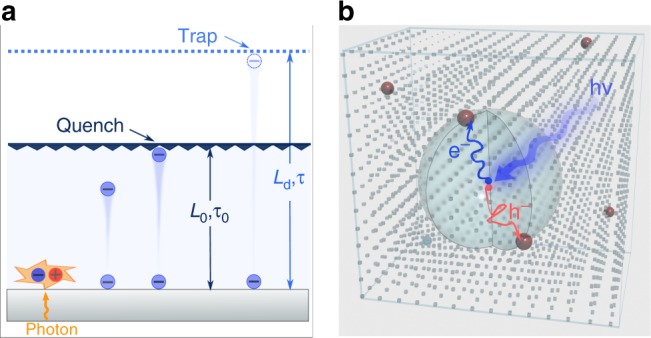
Concept of diffusion-quenching models. **a** 1D diffusion-quenching structure; carrier funneling process occurs at the interface between the active material and carrier extraction layer. **b** Configuration of 3D bulk diffusion-quenching model, with quenching sites (brown spheres) dispersed inside the bulk of material. Diffusion processes of electrons (red curve) and holes (blue curve) occur in all three dimensions (over area of semitransparent large sphere)

Figure 1a illustrates a 1D-diffusion model for an entirely optical method, where a thin film is interfaced with a carrier (electron or hole, electron as an example here) quenching layer. Carriers are photogenerated at the active layer/substrate side, diffuse towards the other side, and then are quenched before recombination in the perovskite takes place. By solving the 1D diffusion equation with the sample thickness (*L*_0_) and quench-limited diffusion time (*τ*_0_), the diffusion coefficient *D* can be acquired. The diffusion length *L*_d_ can therefore be calculated using $L_{\mathrm{d}}^2 = D \cdot \tau$ (Eq. 1), where *τ* stands for the carrier lifetime without the quencher. In this case, a successful fit requires one pair of known parameters (*L*_0_*, τ*_*0*_*, τ*), and also a sample thickness shorter than the diffusion length, (*L*_0_ < *L*_d_).

Thick single-crystal perovskites do not lend themselves to the 1D method, for they are many optical absorption lengths thick. We devised instead a method we term 3D quenching with tunable diffusion distances (Fig. 1b). We utilize homogeneously distributed quenching sites with controllable distance (*L*) inside single crystals, instead of relying on the surface quenching layer used in the 1D method.

In our method, diffusion is investigated in all three dimensions (3D) rather than in one dimension (perpendicular to the quenching layer). *L* can be modulated to be shorter than or longer than *L*_d_ by varying the quenching material concentration. With several pairs of (diffusion distance *L*_*i*_, diffusion time *τ*_*i*_), the diffusion coefficient D can be obtained by fitting to the diffusion equation *D* = *L*_0_^2^/*τ*_*i*_. One can then validate the method by checking whether the fit remains good – i.e. a consistent *L*_d_ is obtained from each [*L*_*i*_,*τ*_*i*_] data set.

From a materials design perspective, though quantum dots inside polycrystalline thin film perovskites have been reported^24^, the study of transport in the absence of perovskite boundaries requires that the dots instead be embedded into perovskite single crystals in a homogenous manner. We needed to develop a synthetic route to incorporate quenching sites into perovskite single crystals in a seamless and homogeneous manner. We pursued a quencher approach that had both type I band alignment with perovskite in order to capture and recombine photocarriers; and a lattice match with the perovskite so that the crystallinity of the perovskite is retained^24^. Lead sulfide (PbS) QDs have been reported to meet the above requirements when epitaxially grown inside methylammonium lead halide perovskite crystals (Supplementary Fig. 1); however, quantum dots have not previously been reported as quenchers applied to study the transport properties of perovskite single crystals.

We first selected the antisolvent vapor-assisted crystallization method to grow perovskite single crystals. The method has previously been shown to produce high quality crystals, a fact attributable to the slow diffusion of the antisolvent into the solvent^5^ (Supplementary Fig. 2a).

Unfortunately, when we added the perovskite capped PbS QDs during the crystallization process, we did not obtain the desired QD-in-single-crystals (QDISCs). Instead, perovskite single crystals in pure phase formed, segregated from the quantum dots.

**Fig. 2:**
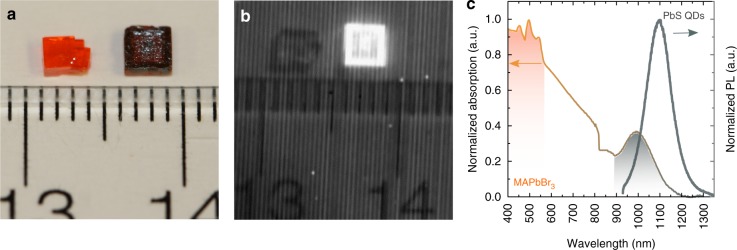
Material platform of quantum dots inside single crystal (QDISCs). **a** Pure MAPbBr_3_ perovskite crystal (left) and QDISCs (right). QDISC exhibit the color of PbS quantum dots. **b** Emission from quantum dots under room light captured by a near-infrared camera. **c** Absorption and photoluminescence spectrum of QDISCs

With the goal of synchronizing the crystallization process with the timescale on which QDs maintain their colloidal stability, we changed our strategy of perovskite crystal synthesis. We sought to shorten the crystallization process to 3 h using instead the inverse temperature crystallization (ITC) method^25^ (Supplementary Fig. 2b). Perovskite single crystals (Fig. 2a, left) grown via the ITC method have been reported to possess trap densities and transport properties on par with the best reported for MAPbBr_3_ crystals to date.

With this strategy, we found that, once PbS QDs were added during crystallization, the resultant crystals exhibited a similar color to that of a QD solution (Fig. 2a, dark crystal on the right).

QDISCs show, at the macroscopic level, the same well-defined cubic shape as do the (dotless) perovskite single crystals (Supplementary Fig. 3). We observed strong infrared luminescence from QDISCs even under room light excitation (Fig. 2b). The optical absorption spectra of QDISCs in Fig. 2c signal the existence of perovskite, with its clear bandedge at around 550 nm, and quantum dots with their excitonic peak at 1000 nm. The steady state photoluminescence spectra of QDISCs show an emission from quantum dots at 1100 nm and, at these dot concentrations, no perovskite emission. The QDs exhibited an absolute photoluminescence efficiency (PLQE) of 24%.

This high PLQE of PbS QDs in the solid state surpasses that observed in the best previous reports^26^. This indicates good passivation by the perovskite and the formation of a type-I heterostructure between QDs and perovskites^27^.

We then turned to study the structural properties of perovskite as a function of QD incorporation. From single crystal XRD measurements, we observed that the crystal structure of the perovskite remains intact following the incorporation of QDs (Supplementary Fig. 3). We then carried out grazing incidence X-ray scattering (GISAXS) measurements on pure perovskite single crystals and QDISCs having high QD loading (60 perovskite:1 QD mass ratio). The samples show the same characteristic scattering pattern (110) of MAPbBr_3_ (Supplementary Fig. 4a and b). The scattering peak full width half maximum (FWHM) of QDISCs closely approaches that of pure perovskite single crystals (0.18 *vs* 0.25 nm^−1^). We ascribe the slightly narrower scattering peak in QDISCs to the reduced rate of sample degradation when quantum dots are incorporated. We conclude that, to within the sensitivity limits of the method, the QDISCs exhibit crystallinity comparable to that of the pure-perovskite single crystals (Supplementary Fig. 4c).

**Fig. 3:**
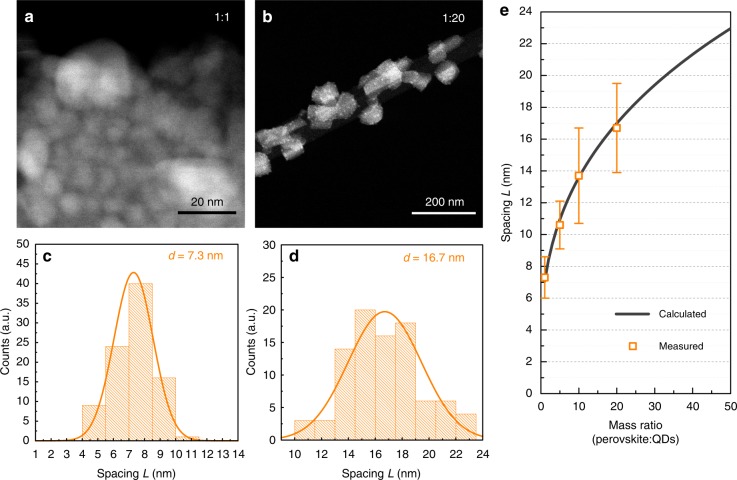
Diffusion distance (*L*) extraction. **a**, **b** STEM images of quantum dots (bright dots) in perovskite single crystals with the mass ratio between QDs and perovskite of 1:1 and 1:20. With the decreasing QD concentration, the inter-dot spacing increases. **c**, **d** Statistic distribution of *L* from over 90 data points from the STEM images. **e** Calculated L as a function of QD concentration (grey line), and experimental data (yellow points). The error bars indicate standard deviation of the measured *L*

To elucidate the interaction between the QDs and perovskite single crystal, we used transmission electron microscopy (TEM) to probe the microscopic structure of QDISCs (Supplementary Fig. 5a–d). TEM images were taken from nanosized QDISCs (Methods). Figure 3 illustrates that quantum dots separate from each other further with decreasing QD concentration (from 1:1 to 1:20 mass ratio). We measured the inter-dot spacing (*L*) for each concentration and plotted the distribution of *L* (Fig. 3c, d and Supplementary Figs. 6–7). We further developed a 3-dimensional homogeneous distribution model to reveal the dependence of *L* on QD concentration (Methods, Supplementary Fig. 8). Figure 5e shows that the experimental data agrees well with the simulated results (with the quantum dots radius equals to 3 nm). We also carried out the time-of-flight secondary ion mass spectrometry (TOF-SIMS) on QDISCs to ascertain whether QDs homogeneously distributed in macroscale single crystals (Supplementary Fig. 9). From these studies, we concluded that the diffusion distance *L* can be accurately modulated by varying the QD concentration.

**Fig. 4:**
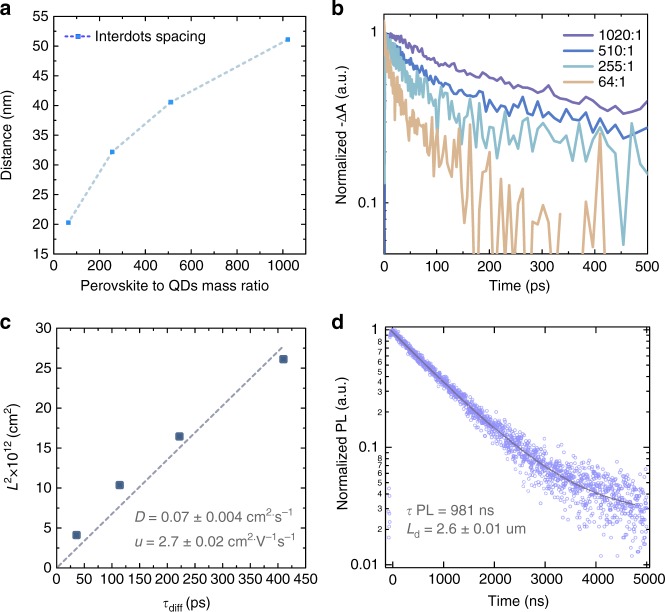
3D diffusion-quenching measurement on MAPbBr_3_ single crystals. **a** Inter-dot spacing tuning via controlling the quantum dot concentration from 64:1 to 1020:1 (mass ratio between perovskite and QDs, QD radius = 2.5 nm). **b** Transient absorption dynamics at perovskite bleach with different diffusion distance (limited by inter-dot spacing). **c** Fitting of diffusion coefficient and carrier mobility of MAPbBr_3_ single crystals. **d** Carrier lifetime and diffusion length calculation of MAPbBr_3_ single crystals

We then proceeded to explore measuring the diffusion length using these heterocrystals. First, we varied the diffusion distance *L*_i_ (which corresponds to the mean value of the Gaussian distribution of inter-dot spacing) from 20 to 50 nm by further decreasing the QD concentration (Fig. 4a). We carried out transient absorption spectroscopy (TAS) to study the carrier diffusion time in QDISCs by probing the dynamics of the perovskite bleach. Figure 4b shows that carrier concentration decays faster when we increase the density of QDs (from 1020:1 to 64:1, perovskite: QDs), and the shorter diffusion time is consistent with the shorter diffusion distance. We estimated the carrier diffusion lifetime (*τ*_diff_) by fitting the TA traces using a monoexponential model. It should be noted that the kinetics was simplified and Auger process may result in the deviation from the single exponential decay, especially under high pumping fluence^28^. We plot it against the diffusion distance *L*_*i*_ in Fig. 4c. The diffusion distance squared (*L*_*i*_^2^) shows a clear linear dependence on the diffusion time (*τ*_diff_), consistent with the application of the diffusion model to the QDISCs material system.

From the slope of the *L*_*i*_^2^-*τ*_diff_ plot, we extract a diffusion coefficient (*D*) for the perovskite of 0.07 cm^2^ s^−1^. From the Einstein relation: *D* *=* *μ*∙*k*_B_∙*T/q* (Eq. 2), where *μ*, *k*_B_, *T* and *q* stand for mobility, Boltzmann’s constant, temperature, and electrical charge, respectively, we calculate a perovskite carrier mobility of 2.7 cm^2^V^−1^s^−1^. The transient absorption lifetime is determined by the type of carriers (electron or hole) that diffuse more slowly. The resulting estimated mobility corresponds thus to that of the transport-limiting carrier^29^.

In order to obtain the diffusion length limited by trap-assisted recombination, we needed to measure the carrier lifetime (τ) of the pristine perovskite single crystals. The photoluminescence lifetime (*τ*_PL_) in MAPbBr_3_ single crystals is limited by non-radiative recombination (which we know from the low PL quantum yield of this material even in its pure phase), so we can estimate the trap-limited carrier lifetime *τ* using transient photoluminescence spectroscopy (Fig. 4d). When we combine the carrier lifetime and diffusion coefficient, we obtain a diffusion length of 2.6 μm, corresponding to a defect density (*N*_trap_) of 6 × 10^10^ cm^−3^.

**Fig. 5:**
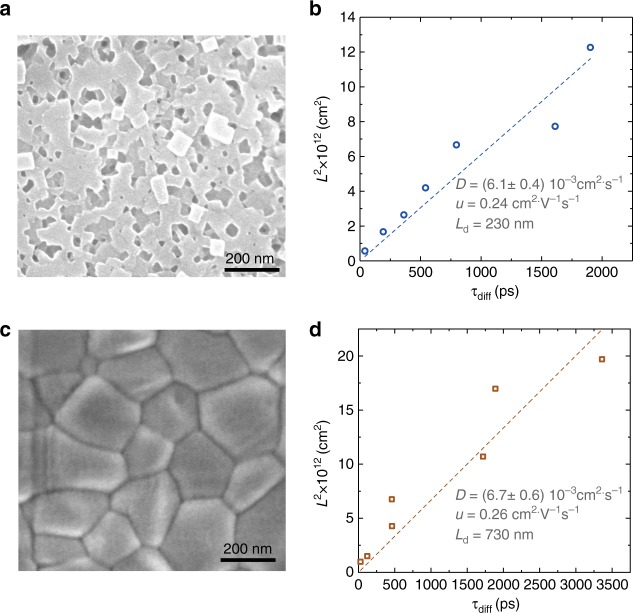
3D diffusion-quenching measurement on perovskite thin films. **a** SEM image of MAPbBr_0.17_I_0.83_ perovskite thin film synthesized using 2-step method. **b** Fitting of diffusion coefficient and carrier mobility of MAPbBr_0.17_I_0.83_ perovskite thin films. **c** SEM image of perovskite thin film with mixed cations and anions (Cs_0.05_MA_0.14_FA_0.81_PbI_2.55_Br_0.45_) synthesized using 1-step method and **d** fitting of coefficient and carrier mobility accordingly

Perovskite thin film absorbers with mixed cations and anions have attracted increasing attention due to their greater efficiency and stability than mono-compositional counterparts^30–33^. We deployed our technique therefore also on thin films based on mixed cation perovskites. We successfully incorporated QDs into both one-step^34^ and two-step^26^ synthesized films with different components: one-step Cs_0.05_MA_0.14_FA_0.81_PbI_2.55_Br_0.45_ and two-step MAPbBr_0.17_I_0.83_ thin films. We varied *L*_*i*_ by changing the QD concentration, and we then fit *L*_*i*_^2^ against *τ*_diff_ (Fig. 5b–d) with a linear function using the same methods as previously discussed in this manuscript. We combined knowledge of the decay times in the pure perovskite films synthesized with different methods (τ = 790 ns and 90 ns respectively) (Supplementary Fig. 10), and estimated a diffusion length of 720 nm (Cs_0.05_MA_0.14_FA_0.81_PbI_2.55_Br_0.45_) and 230 nm (MAPbBr_0.17_I_0.83_), respectively.

Compared to perovskite single crystals, polycrystalline thin films exhibit ten times lower carrier mobilities. As we only probe the diffusion-quenching that occurs within each perovskite grain, we attribute this lower mobility to a greater density of defects within grains, which might be introduced due to the faster crystallization kinetics. Interestingly, we observed similar mobilities between the two thin film perovskites, but the diffusion length of the one-step perovskite with mixed cations and anions (Cs_0.05_MA_0.14_FA_0.81_PbI_2.55_Br_0.45_) is 3 times longer than the two-step perovskite thin film (MAPbBr_0.17_I_0.83_). The results indicate similar bulk defect densities exist in both perovskites, but the surface defect density is much lower in Cs_0.05_MA_0.14_FA_0.81_PbI_2.55_Br_0.45_. This observation agrees well with the fact that the grain size of Cs_0.05_MA_0.14_FA_0.81_PbI_2.55_Br_0.45_ is larger than that of MAPbBr_0.17_I_0.83_ (Fig. 5a–c).

## Discussion

In sum, the present work provides an entirely optical and contactless method to study carrier mobility, lifetime, and diffusion length in perovskite single crystals. The measurement of these parameters in both single crystal and different types of thin film perovskites are poised to contribute to increased understanding and design of high-performance optoelectronic devices.

## Methods

### Quantum dots in perovskite hybrids synthesis

Lead sulfide (PbS) quantum dots were synthesized and then ligand exchanged using previously reported method^26^. Specifically, 3 mL of quantum dots (10 mg mL^−1^) in octane were added into 3 mL dimethylformamide (DMF) solution containing PbI2 (350 mg) and MAI (120 mg). After the mixed solution was stirred for 15 mins, the quantum dots transferred from the top octane solution to the bottom DMF solution. The octane was then removed, followed by three more washes with octane, to completely remove the organic residue. The quantum dots were then precipitated with toluene and dried under vacuum for 10 mins.

After removing the solvent by vacuum, the exchanged quantum dot powder was weighed and then dispersed in the perovskite precursor solution in DMF (MAPbBr_3_ in DMF with 1 M concentration). The crystals of hybrid quantum dots in perovskite formed when slowly increasing the temperature of the solvent from 60 to 120 ℃^11^. The solvent was kept at 120 ℃ for 3 h; then the crystals were collected and dried with nitrogen gas. By changing the mass ratio of quantum dots to perovskite precursor, the interdot spacing can be modulated.

The antisolvent diffusion method was also conducted, by placing a vial of the mixed quantum dots and perovskite precursor crystallization solution in a sealed container, with antisolvent (dichloromethane) inside the container and slowly diffusing into the DMF solution. After several days, the pure crystals of MAPbBr_3_ grew while the quantum dots precipitated at the bottom of the solution, phase separating from the perovskite crystal.

### Photoluminescence measurement

Photoluminescence measurements were performed using a Horiba Fluorolog Time Correlated Single Photon Counting system with photomultiplier tube detectors. A monochromatized Xe lamp and pulsed laser diodes were used as excitation sources for steady-state and transient measurements, respectively. The time resolution of the transient measurements were limited by the instrument response function of approximately 0.13 ns. In order to reduce the effect of reabsorption in the measurement of carrier lifetime of pure perovskite single crystals, we used reflection mode. In reflection mode, most PL signal was generated and collected close to the crystal surface. Absolute PLQE measurements were carried out in a Quanta-Phi integrating sphere according to standard methods published elsewhere^35^, where excitation and emission spectra are measured for the sample directly and indirectly illuminated. The measurements inside integrating sphere takes into account the effect of reabsorption and reflection of the samples.

### TA measurement

A regeneratively amplified Yb:KGW laser (Light Conversion Pharos) was used to generate femtosecond pulses (1030 nm, 5 kHz). A portion of this pulse was passed through an optical parametric amplifier (Light Conversion Orpheus), generating pump pulses of 450 nm light. The pump and remainder of the fundamental were sent into an optical bench (Ultrafast Helios), where the frequency of the pump was halved to 2.5 kHz with an optical chopper. The time delay between pulses was controlled by sending the fundamental through a delay stage; after which, the fundamental was focused onto a near-IR continuum generation crystal (Ultrafast), producing a white-light continuum probe pulse in both the visible and near-IR regions. For the quantum dots in perovskite single crystal samples. the experiments were carried out in reflection mode, where the probe was reflected from the surface of the single crystals and directed toward the detector (Ultrafast Helios). The sample was fixed at the sample holder, and, depending on the strength of the signal, a number of bidirectional scans were averaged to assist with lowering the noise. The power of the pump pulse was set to 11 µW for 450 nm and 225 µW for 1000 nm. For the quantum dots in perovskite thin film samples, the experiments were conducted in transmission mode, with the sample translated at 1 mm/s to reduce the noise.

### TEM measurement

Since the QDISCs are in mm scale, it is difficult to fabricate the ultra-thin (thickness < 50 nm) samples that are suitable for TEM measurement. Alternatively, we turned to synthesize nanosized QDISCs. Specifically, quantum dots in perovskite precursor solution (see details in 1. Quantum dots in perovskite hybrids synthesis) were spin coated onto a plasma-treated TEM grid, with the spin speed of 3000 r.p.m for 60 s. 200 μL of toluene were added during spinning (after 5 s). Then the films were directly transferred to a hotplate and annealed at 90 ℃ for 10 min. All the thin-film fabrication processes were carried out in a N_2_ filled glovebox. For TEM measurement, we synthesized a set of samples with different QD concentration: pure perovskite, QD to perovskite mass ratio of 1:1, 1:5, 1:10, and 1:20.

STEM images were obtained using a Hitachi HF 3300 electron microscope with a cold field emission electron source (energy spread from 0.45 to 0.7 eV) operating at 300 keV and in bright-field (BF) and high-angle annular dark-field (HAADF) modes.

### TOF-SIMS measurement

TOF-SIMS study was conducted by the ToF-SIMS5 from ION-TOF GmbH (Munster, Germany). Samples were measured in a dual beam profiling mode. The primary ion was 30 keV Bi^3+^. This ion beam was applied over a 200 × 200 µm area at the center of a 400 × 400 µm sputter crater. The sputter ion was 1 keV Ar^+^. Spectral data were acquired in a high mass-resolution mode. All profiles were performed in non-interlaced mode.

### Diffusion distance (*L*) calculation and experimental extraction

The inter-dots spacing used in this work is defined as the distance between the center of the nearby two quantum dots which are homogeneously dispersed in a three-dimensional perovskite matrix. In order to quantify the diffusion distance L of the carrier traveling through, which is practically limited by the inter-dots spacing, we developed the following model (Supplementary Fig. 6a). The quantum dots with the radius of r (red spheres) are assumed to be homogeneously dispersed in the perovskite single crystals with the inter-dots spacing of *L*. The densities of PbS quantum dots and MAPbBr_3_ perovskite are $\rho _{QD} = 7.6\,{\rm{g}} \cdot {\rm{cm}}^{ - 3}$ and $\rho _P = 3.5\,{\rm{g}} \cdot {\rm{cm}}^{ - 3}$. Per unit cell of the QDISCs, there is one full quantum dots with the mass of:

1 $$m_{QD} = \rho _{QD} \cdot \frac{4}{3}\pi r^3,$$

and the mass of the perovskite is:

2 $$m_P = \rho _P \cdot \left( {L^3 - \frac{4}{3}\pi r^3} \right).$$

We define the mass ratio between quantum dots and perovskite as:

3 $$\frac{{m_{QD}}}{{m_P}} = \frac{1}{X},$$

with *X* as a variable that can be modulated experimentally to control the inter-dots distance L. Combining Eq. S1–3, we can get the inter-dots spacing as a function of X as below:

4 $$L = r \cdot \root {3} \of {{\frac{4}{3}\pi \left( {\frac{{\rho _{QD}}}{{\rho _P}} \cdot X + 1} \right)}}$$

Supplementary Fig. 6b illustrate the *L*–*X* relation in the cases of using quantum dots with different sizes. In all experiments reported in this work, we used QD with the size of 3 nm.

We used TEM to identify QDs and perovskite matrix: due to the density contrast, QDs correspond to dark dots in bright-field, and bright dots in dark-field (Supplementary Fig. 3a, b). While for pure perovskite crystals, TEM images only reveal contrast from perovskite. For the purpose for the inter-dot spacing measurement, we identified the QDs from the dark-field images.

We prepared 4 different concentrations for nanosized QDISC samples, with the mass ratio between perovskite to quantum dots ranging from 1:1 to 20:1. For each concentration, we collected a data set composing 90 data points of inter-dot spacing with statistical analysis presented next to the TEM images (Supplementary Figs. 4–5). In order to enhance the accuracy of the inter-dots spacing, we only took the thin sample with few tens of nanometer thickness into account. In this way, the 2D projection is closer to the actual space distribution of QDs in 3 dimensional.

## Supplementary information

Supplementary Information

## Data Availability

The data that support the plots within this paper and other findings of this study are available from the corresponding author upon reasonable request.
