# Supplementary material for: Contactless measurements of photocarrier transport properties in perovskite single crystals
Source: Nat Commun. 2019 Apr 8;10:1591. doi: 10.1038/s41467-019-09538-7 (PMC6453944; doi:10.1038/s41467-019-09538-7)
Supplement: Supplementary file 1 — Supplementary Information [file 41467_2019_9538_MOESM1_ESM.pdf]

Supplementary Information

Contactless measurements of photocarrier transport properties in perovskite single crystals

Xiwen Gong[1,†], Ziru Huang[1,†], Randy Sabatini[1], Chih-Shan Tan[1], Golam Bappi[1], Grant Walters[1], Andrew Proppe[1,2], Makhsud I. Saidaminov[1], Oleksandr Voznyy[1], Shana O. Kelley[2,3], Edward H. Sargent[1*]

1. Department of Electrical and Computer Engineering, University of Toronto, 35 St George Street, Toronto, Ontario M5S 1A4, Canada

2. Department of Chemistry, University of Toronto, 80 St. George Street, Toronto, Ontario M5S 1A4, Canada

3. Department of Pharmaceutical Sciences, Leslie Dan Faculty of Pharmacy, University of Toronto, Toronto, Ontario, Canada, M5S 3M2

E-mail: ted.sargent@utoronto.ca

† These authors contributed equally to this work

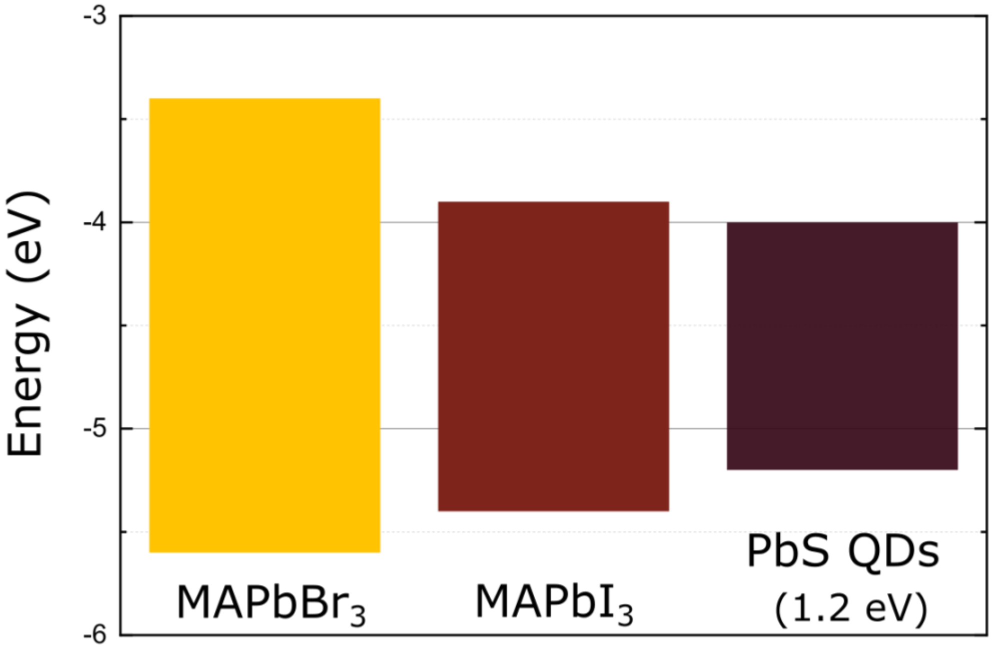

Supplementary Figure 1| Type-I electronic band alignment[1] between perovskites and PbS quantum dots (1.2 eV).

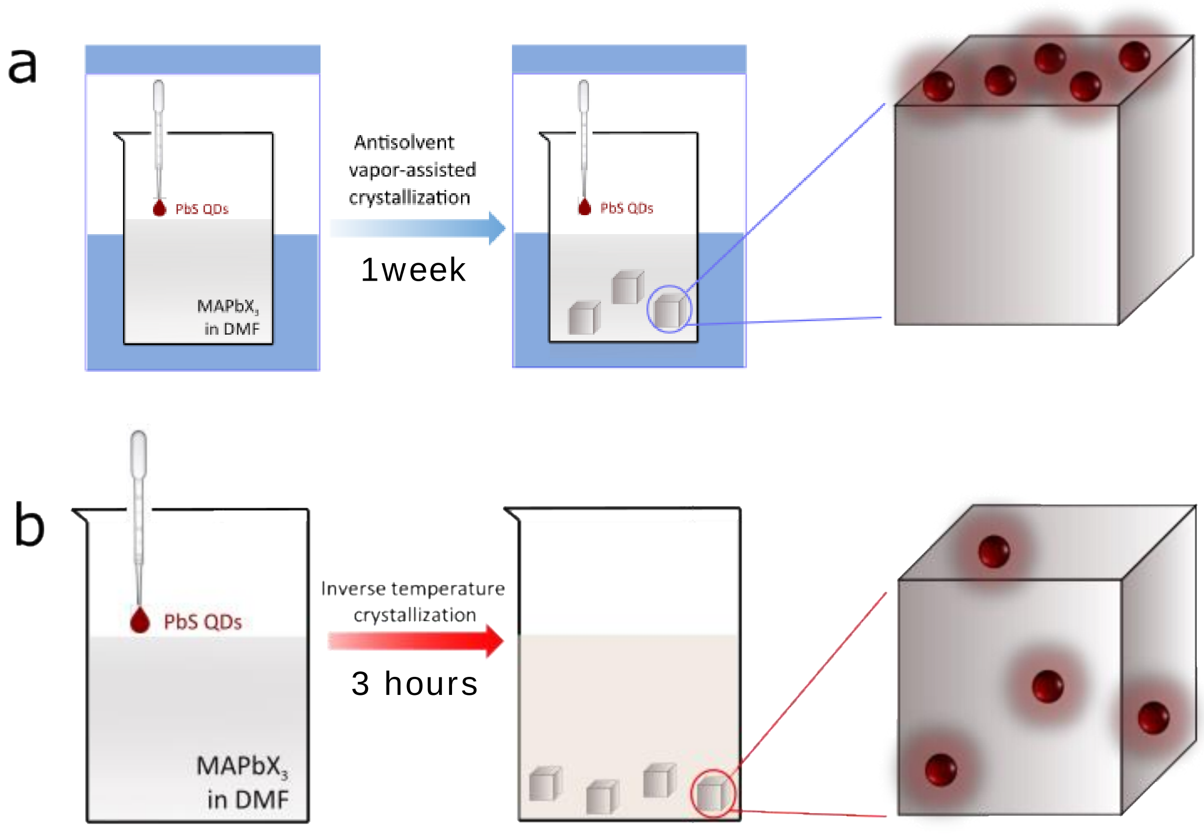

Supplementary Figure 2| Schematic of different synthetic methods. a, Antisolvent vapor-assisted crystallization method, with DMF as good solvent for both QDs and perovskite, and dichloromethane as anti-solvent. The final products show phase-segregation: quantum dots precipitate on top of perovskite single crystals. **b,** Inverse temperature crystallization (ITC) method generate QDISCs with quantum dots homogeneously dispersed inside the perovskite crystals.

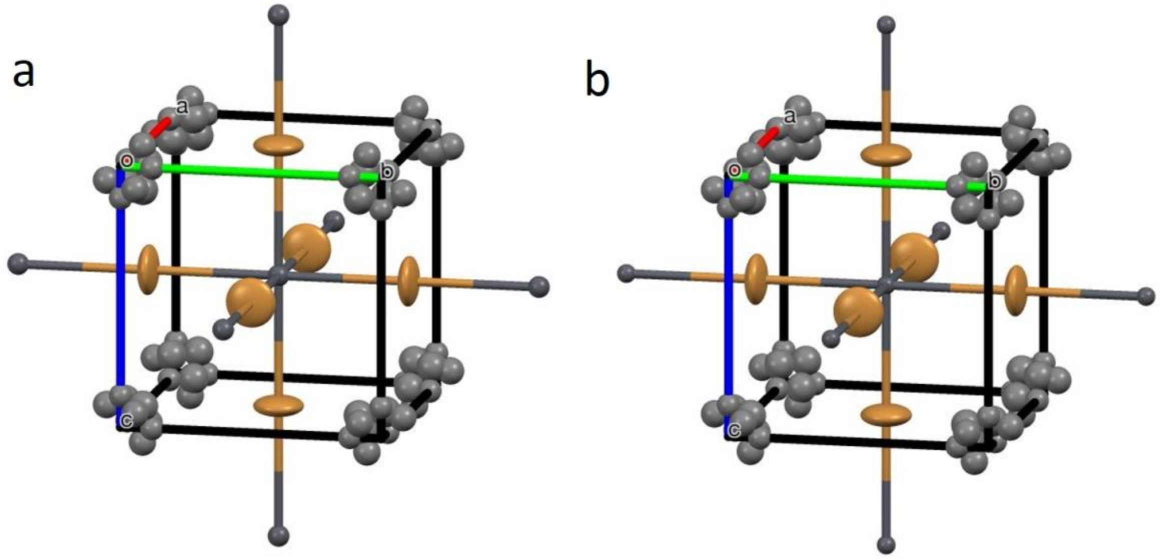

Supplementary Figure 3| Single crystal structure of perovskite single crystals. **a,** Pure $MAPbBr_3$ single crystals. **b,** QDISCs. No obvious change of crystal structure was observed after quantum dot incorporation.

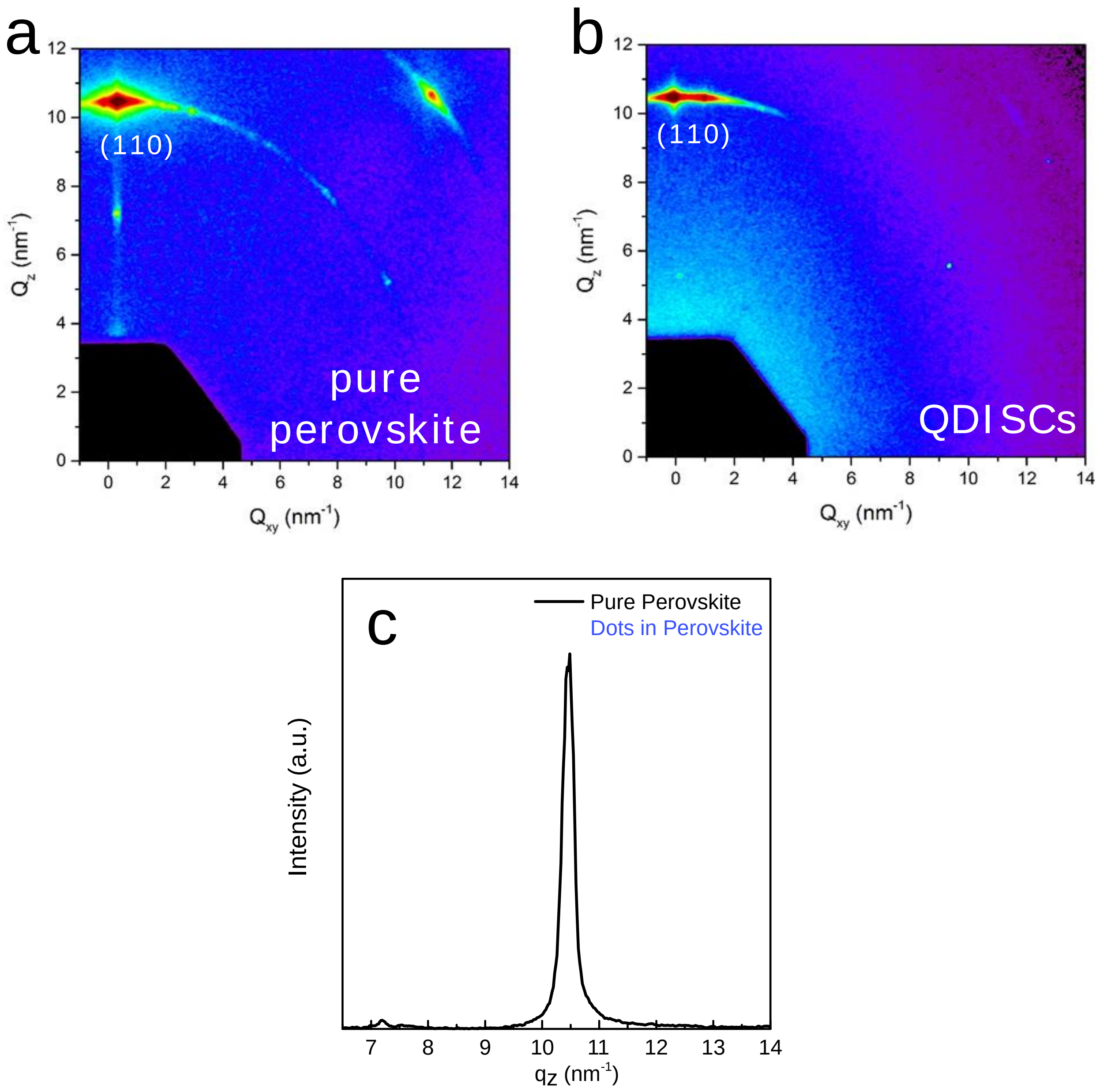

Supplementary Figure 4| Structural information of QDISCs. **a,** GIWAXS patterns of pure MAPbBr3 perovskite single crystal and **b,** QDISCs. **c,** pure perovskite and QDISCs show similar FWHM of the diffraction peak (0.25 vs 0.18 nm^{-1}), indicating the comparable crystallinity of both crystals.

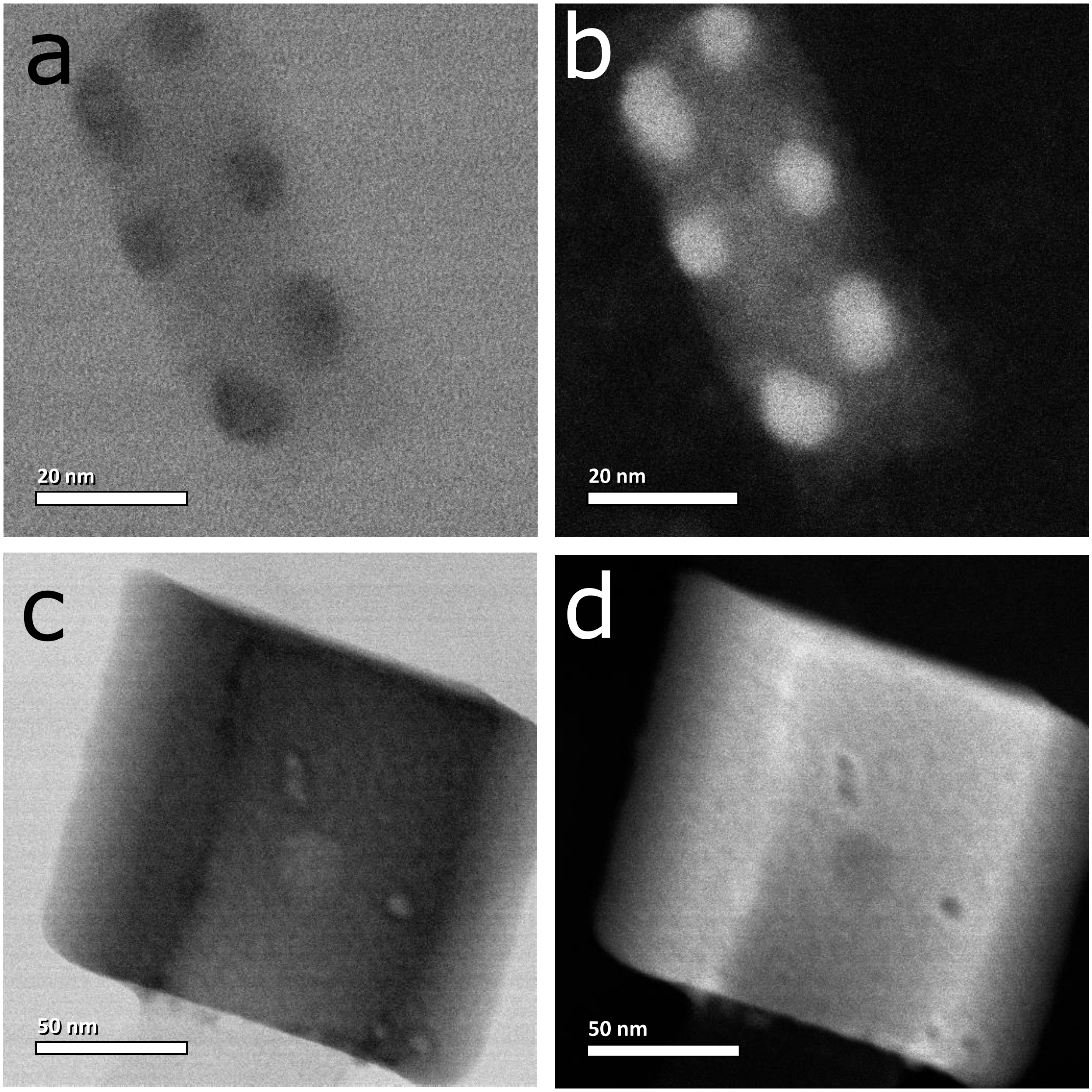

Supplementary Figure 5| TEM and STEM images of QDISCs and pure perovskites. **a,** Bright field TEM image of QDISCs, the PbS QDs exhibit as dark dots due to their higher density. **b,**

STEM image of the same sample as shown in **a.** In the dark field image (STEM mode), quantum dots are brighter dots, due to stronger scattering of the PbS crystal lattice. **c,** TEM image of pure perovskite without QDs and STEM image **d**.

1:5). The data points (~90) were collected from STEM images a-d. The statistic plot is presented in e, with the mean spacing of 10.6 nm.

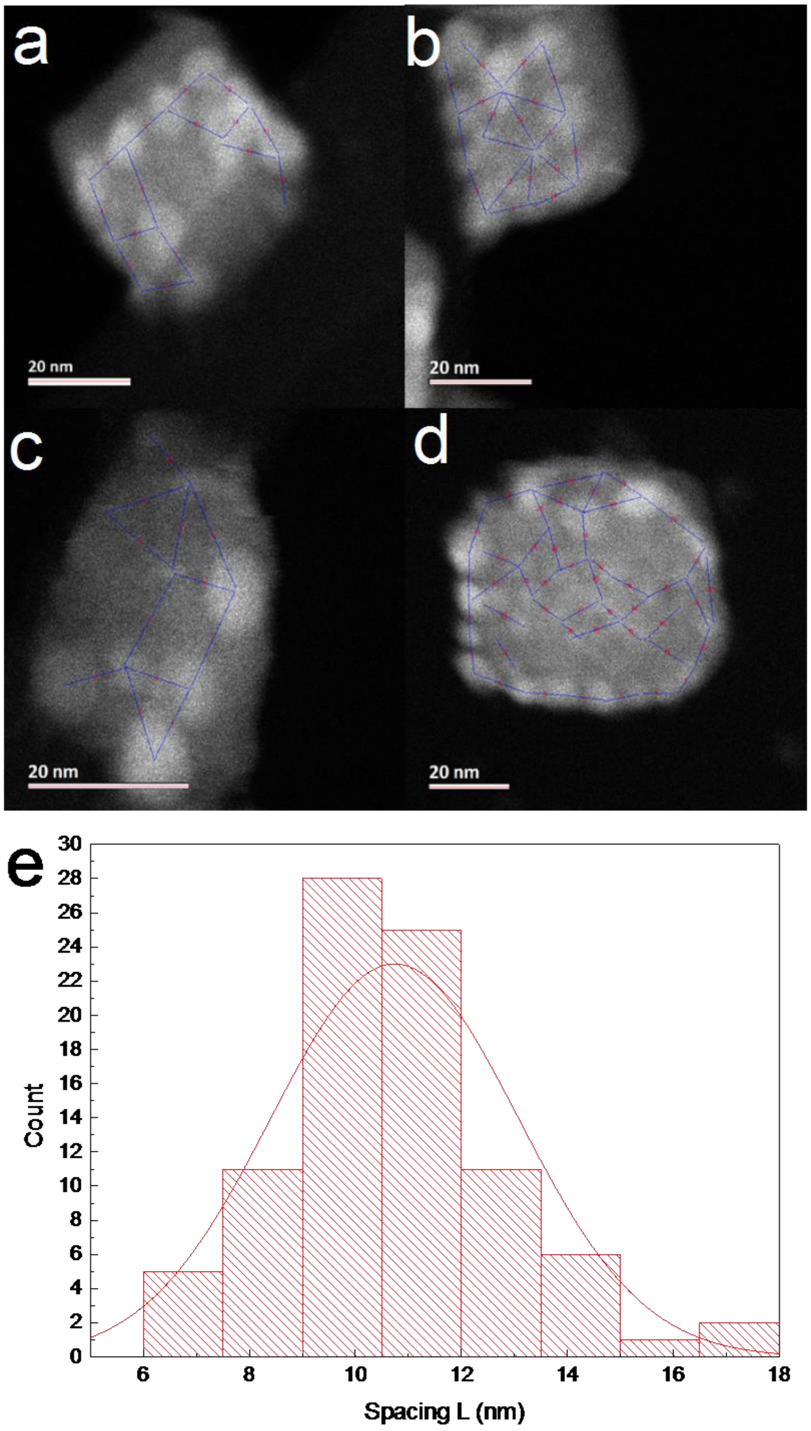

Supplementary Figure 6| Diffusion distance extraction (with QDs: perovskite mass ratio of 1:5). The data points (~90) were collected from STEM images **a-d**. The statistic plot is presented in **e**, with the mean spacing of 10.6 nm.

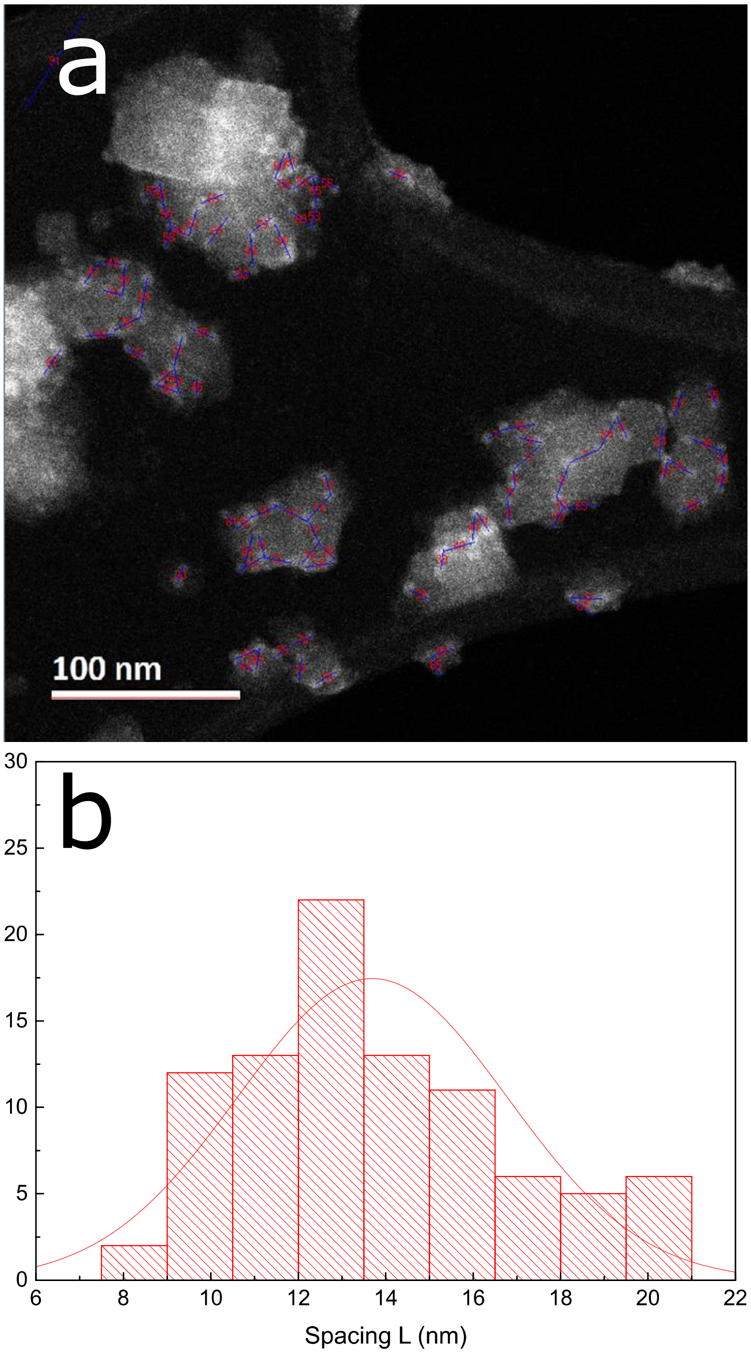

Supplementary Figure 7| Diffusion distance extraction (with QDs: perovskite mass ratio of 1:10). The data points (~90) were collected from STEM images **a,** The statistic plot is presented in **b,** with the mean spacing of 13.7 nm.

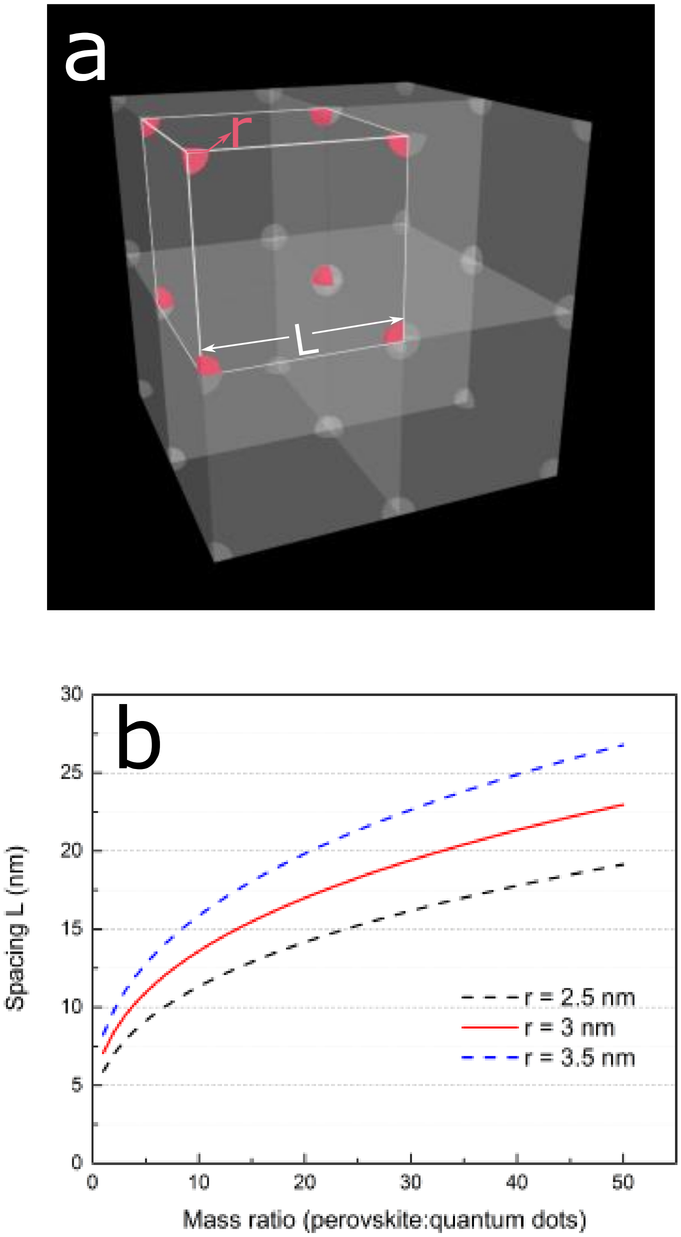

Supplementary Figure 8| 3D homogeneous dispersion model. a, QDs dispersed in perovskite crystals. Each unit cell (side length defined as L) for includes one quantum dots (radius =r) , and the rest of cell is occupied by perovskite. **b,** L as the function of different QD concentration, with the radius of QD equals to 2.5 nm (black dash line), 3 nm (red solid line), and 3.5 nm (blue dash line).

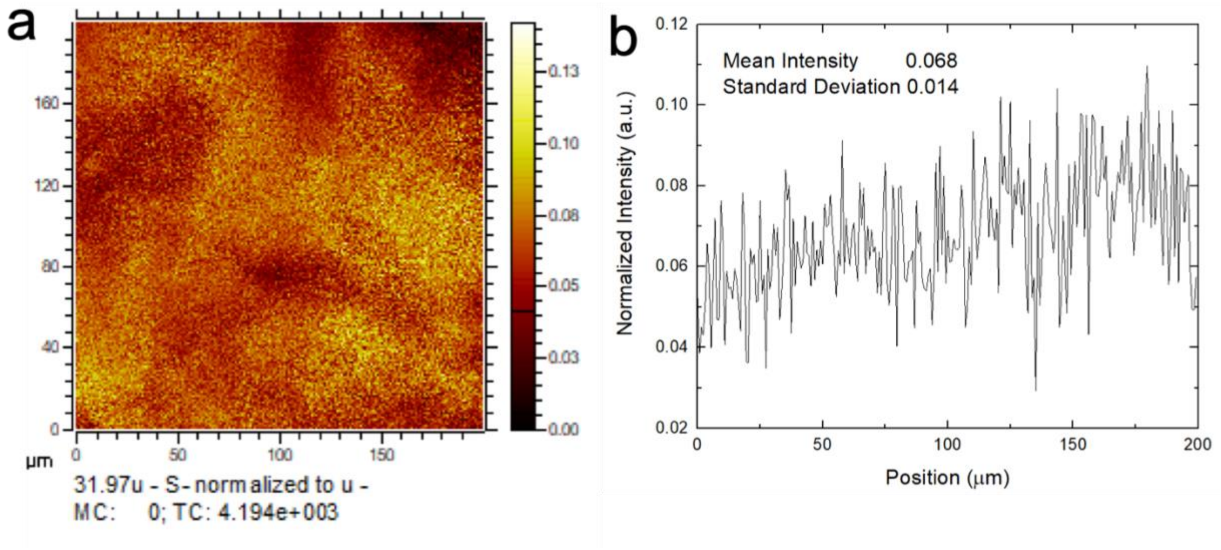

Supplementary Figure 9| TOF-SIMS of QDISC. a, Sulfur mapping of the cross-section of QDISC with the mapping area of 200 μm * 200 μm. **b,** a line scan of the sulfur intensity of Supplementary Figure 8a.

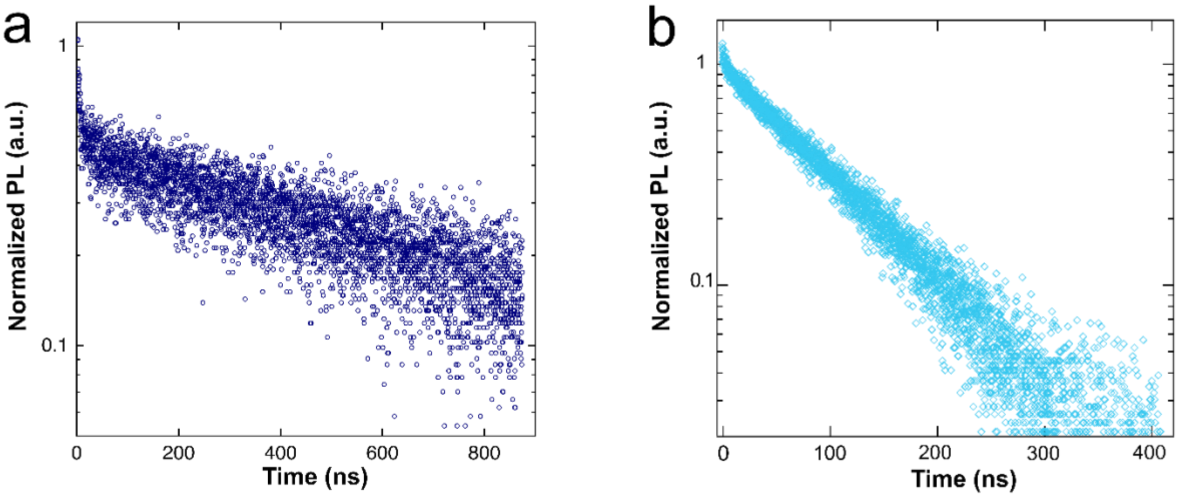

Supplementary Figure 10| Transient PL measurement on pure perovskite thin film. a, pure $Cs_{0.05}MA_{0.14}FA_{0.81}PbI_{2.55}Br_{0.45}$ thin film synthesized via one-step with lifetime of 790 ns. **b,** pure $MAPbBr_{0.17}I_{0.83}$ thin film synthesized via two-step with lifetime of 90 ns.

Supplementary Table 1| Contactless methods used in the measurement of perovskite transport properties.

Reference	Materials	Configuration	Methods	Reported μ ($cm^2 \cdot V^{-1}s^{-1}$)	Reported L_D (μm)
[2]	$CH_3NH_3PbI_3$	Thin films	TRTHz	35	2.5
[3]	$CH_3NH_3PbI_3$	Thin films	TRTHz	8.1	μ m for low charge densities
	$CH_3NH_3PbI_{3-x}Cl_x$			11.6	
[4,5]	$CH_3NH_3PbI_3$	Thin films	TRTHz	20	N/A
[6]	$CH_3NH_3PbI_3$	Thin films	TRMC	30	4
[7]	$CH_3NH_3PbI_3$	Thin films	TRMC	71	N/A
[8]	$CH_3NH_3PbI_3$	Thin films	TRTHz	11	1
[9]	$CH_3NH_3PbI_3$	Thin films	TRMC	29	4.1
[10]	$CH_3NH_3PbI_3$	Thin films	TRTHz	6.2	5
[11]	$CH_3NH_3PbI_3(Cl)$	Thin films	TRTHz	27	N/A
[12]	$CH_3NH_3PbI_3$	Single crystals	TRTHz	550 ~ 800	N/A
[13]	$CH_3NH_3PbI_3$	Single crystals	TRTHz	N/A	0.8~50
[14]	$CH_3NH_3PbI_3$	Single crystals	TRMC	115±15	N/A
[15]	$CH_3NH_3PbI_3$	Thin films	SPV	N/A	N/A
[16]	$CH_3NH_3PbX_3$ (X=I, Br, Cl)	Single crystals	SPV	N/A	N/A

Supplementary References:

1. Morkoç, H. *Nitride Semiconductor Devices : Fundamentals and Applications.* 177-178 (Wiley, 2013).
2. Milot, R. L., Eperon, G. E., Snaith, H. J., Johnston, M. B. & Herz, L. M. Temperature-Dependent Charge-Carrier Dynamics in CH3NH3PbI3 Perovskite Thin Films. *Adv. Funct. Mater.* **25,** 6218–6227 (2015).
3. Wehrenfennig, C., Eperon, G. E., Johnston, M. B., Snaith, H. J. & Herz, L. M. High charge carrier mobilities and lifetimes in organolead trihalide perovskites. *Adv. Mater.* **26,** 1584–1589 (2014).
4. Ponseca, C. S. *et al.* Organometal halide perovskite solar cell materials rationalized: ultrafast charge generation, high and microsecond-long balanced mobilities, and slow recombination. *J. Am. Chem. Soc.* **136,** 5189–92 (2014).
5. Oga, H., Saeki, A., Ogomi, Y., Hayase, S. & Seki, S. Improved understanding of the electronic and energetic landscapes of perovskite solar cells: High local charge carrier mobility, reduced recombination, and extremely shallow traps. *J. Am. Chem. Soc.* **136,** 13818–13825 (2014).
6. Hutter, E. M., Eperon, G. E., Stranks, S. D. & Savenije, T. J. Charge Carriers in Planar and Meso-Structured Organic-Inorganic Perovskites: Mobilities, Lifetimes, and Concentrations of Trap States. *J. Phys. Chem. Lett.* **6,** 3082–3090 (2015).
7. Kim, D. H. *et al.* 300% Enhancement of Carrier Mobility in Uniaxial-Oriented Perovskite Films Formed by Topotactic-Oriented Attachment. *Adv. Mater.* **29,** 1–8 (2017).

8. La-o-vorakiat, C. *et al.* Elucidating the role of disorder and free-carrier recombination kinetics in CH3NH3PbI3 perovskite films. *Nat. Commun.* **6,** 7903 (2015).

9. Reid, O. G., Yang, M., Kopidakis, N., Zhu, K. & Rumbles, G. Grain-Size-Limited Mobility in Methylammonium Lead Iodide Perovskite Thin Films. *ACS Energy Lett.* **1,** 561–565 (2016).

10. Savenije, T. J. *et al.* Thermally activated exciton dissociation and recombination control the carrier dynamics in organometal halide perovskite. *J. Phys. Chem. Lett.* **5,** 2189–2194 (2014).

11. Karakus, M. *et al.* Phonon-Electron Scattering Limits Free Charge Mobility in Methylammonium Lead Iodide Perovskites. *J. Phys. Chem. Lett.* **6,** 4991–4996 (2015).

12. Valverde-Chávez, D. A. *et al.* Intrinsic femtosecond charge generation dynamics in single crystal CH3NH3PbI3. *Energy Environ. Sci.* **8,** 3700–3707 (2015).

13. Bi, Y. *et al.* Charge Carrier Lifetimes Exceeding 15 μs in Methylammonium Lead Iodide Single Crystals. *J. Phys. Chem. Lett.* **7,** 923–928 (2016).

14. Semonin, O. E. *et al.* Limits of Carrier Diffusion in n-Type and p-Type CH3NH3PbI3Perovskite Single Crystals. *J. Phys. Chem. Lett.* **7,** 3510–3518 (2016).

15. Barnea-Nehoshtan, L., Kirmayer, S., Edri, E., Hodes, G. & Cahen, D. Surface Photovoltage Spectroscopy Study of Organo-Lead Perovskite Solar Cells. *J. Phys. Chem. Lett.* **5,** 2408–2413 (2014).

16. Liu, X., Liu, Y., Gao, F., Yang, Z. & Liu, S. Photoinduced surface voltage mapping study for large perovskite single crystals. *Appl. Phys. Lett.* **108,** 1–6 (2016).
